# A methodology for utilization of predictive genomic signatures in FFPE samples

**DOI:** 10.1186/1755-8794-4-58

**Published:** 2011-07-11

**Authors:** Jennifer A Freedman, Christina K Augustine, Angelica M Selim, Kirsten C Holshausen, Zhengzheng Wei, Katherine A Tsamis, David S Hsu, Holly K Dressman, William T Barry, Douglas S Tyler, Joseph R Nevins

**Affiliations:** 1 Institute for Genome Sciences and Policy, Duke University Medical Center, DUMC Box 3382, Durham, 27708, USA; 2 Department of Surgery, Duke University Medical Center, DUMC Box 3704, Durham, 27710, USA; 3 Department of Medicine, Duke University Medical Center, DUMC Box 3230, Durham, 27710, USA; 4 Department of Biostatistics and Bioinformatics, Duke University Medical Center, DUMC Box 2721, Durham, 27710, USA; 5 Department of Molecular Genetics and Microbiology, Duke University Medical Center, DUMC Box 3054, Durham, 27710, USA; 6 Department of Pathology Clinical Services, Duke University Medical Center, DUMC Box 3712, Durham, 27710, USA

## Abstract

**Background:**

Gene expression signatures developed to measure the activity of oncogenic signaling pathways have been used to dissect the heterogeneity of tumor samples and to predict sensitivity to various cancer drugs that target components of the relevant pathways, thus potentially identifying therapeutic options for subgroups of patients. To facilitate broad use, including in a clinical setting, the ability to generate data from formalin-fixed, paraffin-embedded (FFPE) tissues is essential.

**Methods:**

Patterns of pathway activity in matched fresh-frozen and FFPE xenograft tumor samples were generated using the MessageAmp Premier methodology in combination with assays using Affymetrix arrays. Results generated were compared with those obtained from fresh-frozen samples using a standard Affymetrix assay. In addition, gene expression data from patient matched fresh-frozen and FFPE melanomas were also utilized to evaluate the consistency of predictions of oncogenic signaling pathway status.

**Results:**

Significant correlation was observed between pathway activity predictions from paired fresh-frozen and FFPE xenograft tumor samples. In addition, significant concordance of pathway activity predictions was also observed between patient matched fresh-frozen and FFPE melanomas.

**Conclusions:**

Reliable and consistent predictions of oncogenic pathway activities can be obtained from FFPE tumor tissue samples. The ability to reliably utilize FFPE patient tumor tissue samples for genomic analyses will lead to a better understanding of the biology of disease progression and, in the clinical setting, will provide tools to guide the choice of therapeutics to those most likely to be effective in treating a patient's disease.

## Background

Gene expression profiling continues to contribute to advances in clinical oncology, providing a basis for understanding the complex biology of tumors, improving the accuracy of disease diagnosis as well as disease prognosis, and providing tools to determine which targeted therapeutic agents are likely to be effective in the treatment of particular tumors. While the majority of studies have made use of fresh tissue samples so as to optimize the measurement of gene expression, an ability to generate reliable and consistent data from formalin-fixed, paraffin-embedded (FFPE) tissue samples has several advantages. First, FFPE tissue samples are readily available in large numbers across multiple stages of disease and thus the capability to utilize FFPE tissue samples broadens the scope of potential studies. Second, utilization of FFPE tissue samples allows profiling of archived samples for which patient outcomes are already known. Third, utilization of FFPE tissue samples allows profiling of samples from cancers for which all tissue samples are FFPE after examination of clinicopathologic characteristics, such as melanoma samples undergoing an assessment of the prognostic factor of Breslow tumor thickness, which is most accurately measured using the entire tumor obtained from an excisional biopsy.

Several studies have investigated methods to facilitate gene expression profiling from FFPE tissues (for review see [1]). Good correlations have been observed in gene expression profiles from fresh-frozen and FFPE lipopolysaccharide-stimulated human bone marrow stromal cells [2]. With respect to human tumors, concordance has been found between gene expression profiles from fresh-frozen and FFPE colonic epithelial cells isolated by laser capture microdissection [3]. In addition, studies have shown significant overlap between differentially expressed genes in normal versus cancerous colon and breast fresh-frozen and FFPE tissues, in fresh-frozen and FFPE lymphoma and carcinoma, and in FFPE BRCA1 mutant versus sporadic breast cancers [4-6]. Furthermore, studies have generated predictive models from FFPE tissues, including a genomic profile of nontumoral liver tissue surrounding hepatocellular carcinoma that correlates with survival and of primary extremity soft tissue sarcoma that correlates with metastatic recurrence [7,8]. Finally, concordance has been observed between unsupervised hierarchical clusters of gene expression data and tumor type of FFPE carcinomas and the tissue of origin of 3 unknown carcinomas has been elucidated [9].

We have previously described methods to generate gene expression signatures reflecting the activity of a number of oncogenic signaling pathways [10,11]. These pathway gene expression signatures have been used to predict the status of the respective pathways in mouse as well as human tumors. The opportunity to use these signatures to dissect the complexity of tumors, rather than simply using global expression data across >30 k genes, provides not only a more in-depth understanding of tumor subtypes, but also reveals opportunities for novel therapeutic strategies in subgroups of patients, as this approach has been shown to predict sensitivity to various cancer drugs that target components of the relevant pathway [10,12]. Given the need to develop tools that can be applied in a clinical setting, we have focused on developing the capability to apply these same pathway analyses to more readily available FFPE tissue samples.

## Methods

### Generation of paired fresh-frozen and FFPE xenografts

Six week old female nude mice were injected subcutaneously into the lower right abdominal regions near the hind limb with 4 to 7 million human-derived metastatic melanoma cell lines suspended in a 2:1 mix of phosphate buffered saline (PBS) and Matrigel basement membrane. All animal protocols were approved by the Duke University Medical Center Institutional Animal Care and Use Committee. The melanoma cell lines used (DM443, DM440, DM366, DM738 and DM6) were kindly provided by Dr. Hilliard Seigler (Duke University Medical Center) and were confirmed mycoplasma and pathogen-free prior to animal studies. Each cell line was injected into five mice generating a total of 25 xenografts. Tumors were allowed to grow until approximately 1000 mm^3 ^(10 to 15 mm in diameter; two to four weeks) at which time they were harvested, cleaned of surrounding connective tissue and skin, divided into 2 pieces and immediately snap frozen or placed in paraformaldehyde (4% solution in PBS; USB 19943) and fixed overnight at 4°C.

### Human melanoma samples

Eight-10 μm sections were obtained from each patient's FFPE block banked at the Department of Pathology at Duke University Medical Center. All patients were enrolled after obtaining written informed consent and tissue samples were collected according to a protocol approved by the Duke University Medical Center Institutional Review Board.

### RNA preparation

Snap frozen, fresh tissue was homogenized using Lysing Matrix A (MP Biomedicals) and a mini bead-beater (Biospec Products) and RNA was isolated using the RNeasy kit (Qiagen). Fixed tissue was paraffin-embedded and RNA was isolated from eight-10 μm FFPE sections. RNA was isolated using the RecoverAll-MagMAX Custom Kit and protocol (Applied Biosystems), with the following modifications: RNA isolation digestions were incubated at 50°C for 15 minutes and then 80°C for 15 minutes, Lysis Binding Solution was reconstituted using 22 ml of 100% isopropanol (Mallinckrodt Chemicals), Wash Solution 1H was reconstituted using 12 ml of 100% isopropanol (Mallinckrodt Chemicals), and Wash Solution 2 was reconstituted using 44 ml of 100% ethanol (Pharmco-Aaper).

### DNA microarray analysis

RNA was amplified according to the Affymetrix One-Cycle (Affymetrix, Santa Clara, CA) or the MessageAmp Premier protocol (Ambion). Affymetrix DNA microarray analysis was prepared according to the manufacturer's instructions, and targets were hybridized to the Human U133A 2.0 GeneChip (Affymetrix, Santa Clara, CA). All microarray data are available at http://data.genome.duke.edu/Freedman_CEL_Files and on GEO (GSE29598).

### Pre-processing of microarray data

CEL files were RMA normalized using the normalize.R script (available at http://data.genome.duke.edu/Freedman_CEL_Files) run in R (ver2.6.0). CEL files were MAS5.0 normalized using Expression Console Version 1.1 (Affymetrix). All subsequent statistical analyses were performed in R/Bioconductor, Partek, MATLAB, and Eisen's cluster softwares.

### Unsupervised clustering

Hierarchical clustering was performed using Cluster 3.0. The MAS5.0 normalized data was imported into Cluster 3.0. Data was filtered using the SD (Gene Vector) property resulting in a dataset containing 1000 genes. The filtered data was then mean centered for genes and arrays. Clustering of the adjusted data, genes and arrays, was done using the correlation (uncentered) similarity metric and average linkage clustering. The heatmap and dendogram were visualized using Java TreeView 1.0.12. Principal components analysis was performed on whole-genome expression data.

### Pathway analysis

The experimental design and statistical models used to generate patterns of pathway activity in the xenografts and melanomas were done as previously described [10,11]. Gene expression signatures to measure the activity of the RAS and MYC signaling pathways were built using a Bayesian probit regression model for 'metagene' factors from a singular value decomposition of the top differentially expressed genes. A Monte Carlo Markov Chain (MCMC algorithm) was used to generate the predicted probabilities of pathway activity in normalized investigational samples. See Additional file 1 for greater detail of pathway analyses.

## Results and discussion

The development of genomic signatures reflecting the activation of cell signaling pathway activity has been shown to have value in dissecting tumor heterogeneity [10,11] as well as providing a means to direct the use of pathway-specific therapies [10,12]. Nevertheless, it has proven to be difficult to generate robust measures with genome-wide assays such as DNA microarrays using the degraded RNA from FFPE samples. Recent work has described a methodology (MessageAmp Premier) that has potential to generate useful data from these samples. Here we focus on an analysis of the capacity of this methodology to generate consistent biological information from FFPE samples that is concordant with results generated using traditional fresh-frozen samples and a standard Affymetrix assay. Our strategy makes use of a collection of precisely matched fresh-frozen and FFPE tumor tissue samples for validation.

### Generation of paired fresh-frozen and FFPE xenograft samples

In order to generate paired fresh-frozen and FFPE xenograft samples from which gene expression data could be obtained for comparative purposes, we made use of human melanoma-derived cell lines grown as xenografts in a murine model. Human melanoma cells were injected subcutaneously into the right hind limb of six week old female nude mice. Five unique human melanoma-derived cell lines were injected into five mice each (~5 × 10^6 ^cells/injection) generating 25 xenografts. The tumors were allowed to grow to 10 to 15 millimeters in diameter (12 to 33 days) at which point tumors were harvested and skin removed. Each tumor was divided with one-half of each tumor snap frozen while the remaining half was formalin-fixed, paraffin-embedded.

**Figure 1:**
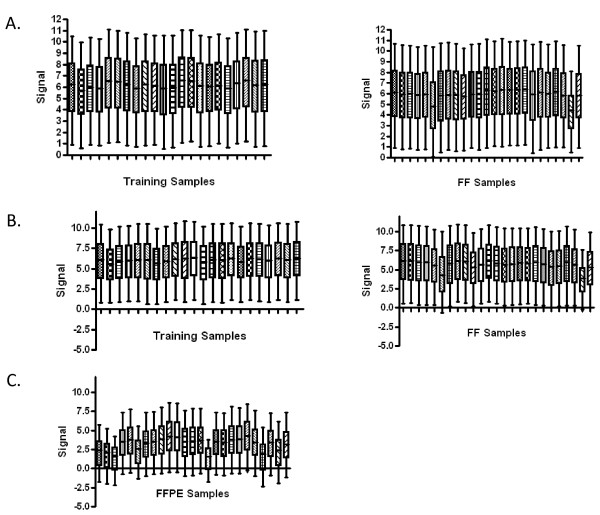
**Distribution of signal intensities**. (A) Training samples and fresh-frozen xenografts processed according to the Affymetrix One-Cycle protocol (B) training samples and fresh-frozen xenografts processed according to the MessageAmp Premier protocol (C) FFPE xenografts processed according to the MessageAmp Premier protocol. For each sample, the 5^th^, 25^th^, 50^th^, 75^th^, and 95^th ^percentile of the MAS5.0 normalized signal values for all probes on the microarray was calculated. The resulting number was divided by the scaling factor. The logarithm to the base 2 of the resulting quotient was determined. These numbers are visualized by the box-and-whisker plots.

RNA from the fresh-frozen xenograft samples, as well as a collection of cells infected with recombinant adenoviruses that are used as the source of training data to develop signatures, was amplified according to the standard Affymetrix One-Cycle protocol and hybridized to Affymetrix Human Genome U133A 2.0 arrays. The quality control metrics for the training samples and fresh-frozen xenograft samples are consistent with the general experience of quality measures (Table 1). Average background values were comparable across all samples and were within the 20-100 normal range. The ratios of the signal values of the 3' probe sets compared to the corresponding 5' probe sets for β-actin were at or below 3 for 24 of 25 samples (ranging from 0.46-3.3). Similar results were obtained for GAPDH, with all samples exhibiting ratios below 3 (ranging from 0.94-1.67). Percent present calls ranged from 49-62% for 24 of 25 samples. Scaling factors were below 3-fold for 23 of 25 samples. As shown in Figure 1A, a box plot of the log signal intensities shows that samples exhibit consistent distributions of signal intensities.

**Table 1:** Quality control metrics for training and fresh-frozen xenograft samples processed using the Affymetrix One-Cycle protocol

	Average Background Value	3' to 5' Ratio β-actin	3' to 5' Ratio GAPDH	Percent Present	Scaling Factor
Training Samples	39.9-53.3	1.3-7.21	0.94-1.27	58.1-62.3	1.4-3.62

Fresh-Frozen Xenograft Samples	38.9-54.4	0.46-2.90	0.94-1.67	33.6-59.9	1.4-9.58

The quality control metrics for the RNA from training samples and fresh-frozen xenograft samples amplified according to the MessageAmp Premier protocol and hybridized to Affymetrix Human Genome U133A 2.0 arrays were similar to those obtained for the RNA amplified according to the Affymetrix One-Cycle protocol (summarized in Table 2). As shown in Figure 1B, a box plot of the log signal intensities shows that all samples exhibit consistent distributions of signal intensities.

**Table 2:** Quality control metrics for training and fresh-frozen xenograft samples processed using the Ambion Message Amp Premier protocol

	Average Background Value	3' to 5' Ratio β-actin	3' to 5' Ratio GAPDH	Percent Present	Scaling Factor
Training Samples	33.4-43.3	1.21-8.24	0.92-2.36	58-62.8	1.77-5.74

Fresh-Frozen Xenograft Samples	33-41.5	0.68-5.17	1.01-1.76	36.5-61.6	1.63-14.3

In contrast, the quality control metrics obtained for the RNA isolated from FFPE xenograft samples, amplified according to the MessageAmp Premier protocol and hybridized to Affymetrix Human Genome U133A 2.0 arrays, were indicative of poorer RNA sample quality (Table 3). Average background values for all FFPE xenograft samples were within the 20-100 normal range and were comparable across all samples. Indicative of more degraded RNA, the ratios of the signal values of the 3' probe sets compared to the corresponding 5' probe sets for β-actin and GAPDH were above 3 for all FFPE xenograft samples, ranging from 6.32-248. Again indicative of poorer sample quality, percent present calls for the FFPE xenograft samples ranged from 8.67-49.7%, with an average percent present call of 37%. Scaling factors for FFPE xenograft samples were all above 3-fold also indicating the existence of more degraded RNA. As shown in Figure 1C, a box plot of the log signal intensities shows that the FFPE xenograft samples exhibit a more inconsistent distribution of signal intensities, exhibiting lower signal intensities and more variation in signal intensities compared to both cell line training and fresh-frozen xenograft samples.

**Table 3:** Quality control metrics for FFPE xenograft samples processed using the Ambion Message Amp Premier protocol

	Average Background Value	3' to 5' Ratio β-actin	3' to 5' Ratio GAPDH	Percent Present	Scaling Factor
FFPE Xenograft Samples	28.7-36	6.32-248	19.1-176	8.67-49.7	7.52-121

**Figure 2:**
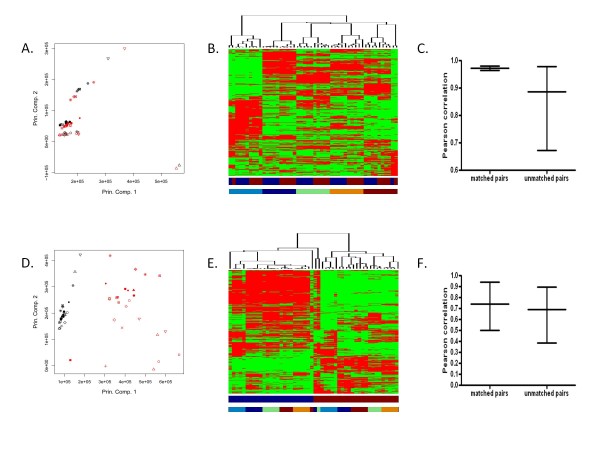
**Patterns of whole-genome expression among paired samples**. (A) Principal component analysis of the MAS5.0 normalized gene expression data from the fresh-frozen xenografts processed with the Affymetrix One-Cycle versus MessageAmp Premier protocol. Shapes represent samples; Affymetrix One-Cycle in black and MessageAmp Premier in red. (B) Unsupervised hierarchical clustering of the MAS5.0 normalized gene expression data from fresh-frozen xenografts processed with the Affymetrix One-Cycle versus MessageAmp Premier protocol. Columns represent samples; rows represent genes; higher expression in red and lower in green. Color bar below data matrix defines Affymetrix One-Cycle in blue and MessageAmp Premier in red. Second color bar defines samples associated with DM6 in dark blue, DM366 in light blue, DM440 in green, DM443 in orange, and DM738 in red. (C) Pearson correlation coefficients for whole-genome expression data from matched and unmatched pairs of fresh-frozen xenografts processed according to the Affymetrix One-Cycle versus MessageAmp Premier protocol. (D) Principal component analysis of the MAS5.0 normalized gene expression data from fresh-frozen xenografts versus FFPE xenografts processed with the MessageAmp Premier protocol. Shapes represent samples; fresh-frozen in black and FFPE in red. (E) Unsupervised hierarchical clustering of the MAS5.0 normalized gene expression data from fresh-frozen xenografts versus FFPE xenografts processed with the MessageAmp Premier protocol. Representations are as in (B), except the color bar below the data matrix defines fresh-frozen samples in blue and FFPE samples in red. (F) Pearson correlation coefficients for whole-genome expression data from matched and unmatched pairs of fresh-frozen xenografts versus FFPE xenografts processed with the MessageAmp Premier protocol.

To evaluate the concordance of whole-genome expression profiles between arrays processed according to the Affymetrix One-Cycle protocol and according to the MessageAmp Premier protocol, unsupervised principal component analysis and hierarchical clustering were used to visualize the level of similarity among pairs from 25 fresh-frozen xenograft samples. As shown in Figure 2A, principal component analysis (PCA) plot shows strong similarity in the whole-genome expression data for a given fresh-frozen xenograft sample processed according to the Affymetrix One-Cycle protocol as compared to the MessageAmp Premier protocol (for principal component analyses in greater than two dimensions and a graph depicting the percentage of variance that each principal component captures see Additional file 2). In addition, a heatmap with hierarchical clustering of fresh-frozen xenograft samples demonstrates the gene expression patterns observed within xenograft sample pairs processed according to the two methods (Figure 2B). Furthermore, the average Pearson correlation coefficient (0.973, range [0.965, 0.981]) for whole-genome expression data obtained from samples processed according to the Affymetrix One-Cycle protocol versus the MessageAmp Premier protocol show much greater agreement than among unmatched pairs (0.886, range [0.673, 0.978]) (Figure 2C) (for a heat map representing the correlation coefficients see Additional file 3).

The same analyses were performed to assess the level of correlation between whole-genome expression data obtained from paired fresh-frozen and FFPE xenograft samples processed according to the MessageAmp Premier protocol. As shown in Figure 2D, PCA plot indicates the whole-genome expression data for a given fresh-frozen xenograft sample is different than the whole-genome expression data for the paired FFPE xenograft sample (for principal component analyses in greater than two dimensions and a graph depicting the percentage of variance that each principal component captures see Additional file 4). In addition, a heatmap with hierarchical clustering of fresh-frozen and FFPE xenograft samples shows that clustering is driven by whether a xenograft sample is of fresh-frozen or FFPE origin rather than by differences in gene expression data between different individual xenografts (Figure 2E). Furthermore, Pearson correlation coefficients measured within a given pair of fresh-frozen versus FFPE xenograft samples are no higher than across unmatched samples (Figure 2F) (for a heat map representing the correlation coefficients see Additional file 5).

### Predictive capacity of pathway signatures in fresh-frozen and formalin fixed, paraffin-embedded xenograft samples

Although whole-genome expression data correlates poorly between paired fresh-frozen and FFPE xenograft samples, the critical question is the extent to which useful and consistent information about the underlying biology can be obtained from the FFPE samples. We have previously made use of gene expression signatures developed to measure the activity of a number of oncogenic signaling pathways to explore the underlying biology of tumor samples [10,11,13]. At the same time, these pathway signatures have been shown to predict sensitivity to various cancer drugs that target components of the relevant pathway and thus provide the further benefit of potentially identifying therapeutic options for subgroups of patients [10,12]. We predicted the activity of the RAS and MYC pathways in the paired fresh-frozen and FFPE xenograft samples, leading to the generation of probability measures that have been shown in previous work to reflect the state of pathway activity as measured by various biochemical assays.

To obtain total RNA for training data, human mammary epithelial cells (HMECs) were infected with a recombinant adenovirus containing either a control insert expressing GFP (eight replicates), an insert expressing RAS (eight replicates), or an insert expressing MYC (six replicates). Total RNA isolated from these cells was amplified using the MessageAmp Premier protocol. For comparison, total RNA isolated from these cells was also amplified using the Affymetrix One-Cycle protocol. Similarly, for the fresh-frozen xenograft samples, total RNA isolated from the tumors was amplified using the MessageAmp Premier protocol and, for comparison, the total RNA isolated from the tumors was also amplified using the Affymetrix One-Cycle protocol. For the FFPE xenograft samples, total RNA isolated from the tumors was amplified using the MessageAmp Premier protocol. All amplified RNA was hybridized to Affymetrix Human Genome U133A 2.0 arrays.

**Figure 3:**
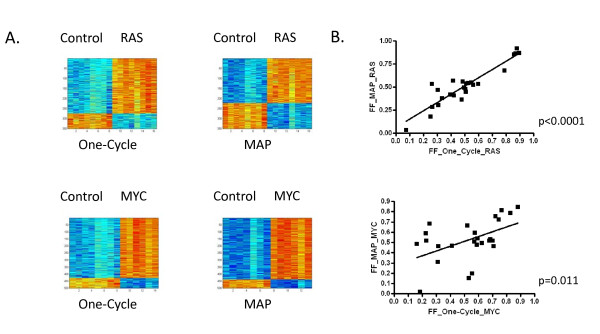
**Status of RAS and MYC pathway activities in fresh-frozen xenografts processed according to the Affymetrix One-Cycle protocol compared to the MessageAmp Premier protocol**. (A) Heatmaps representing the expression levels of genes comprising the gene expression signature that differentiates between HMECs infected with adenovirus expressing GFP (control) versus HMECs infected with adenovirus expressing RAS or MYC as indicated. Within the heatmap, columns represent samples and rows represent genes, with expression values on a low to high continuum represented by a blue to red continuum, respectively. (B) Scatterplot of the Affymetrix One-Cycle protocol (X-axis) versus the MessageAmp Premier protocol (Y-axis) for the RAS pathway (top) or MYC pathway (bottom). The simple linear regression model and p-value is drawn for each Pearson correlation.

For comparison, to build the predictive models from whole-genome expression data we fit a Bayesian regression model to metagene factors of differentially expressed genes of RAS and MYC pathway activity in HMECs. The models are then used to predict the activation status in fresh-frozen xenograft samples processed according to the Affymetrix One-Cycle protocol versus the MessageAmp Premier protocol. As shown in Figure 3A, predictors from either protocol consisting of 350 genes or 500 genes were selected to differentiate between HMECs expressing RAS and MYC, respectively, relative to HMECs expressing GFP. Probes are provided in Additional file 6 for each signature. The Bayesian models were then used to predict the probability of RAS and MYC pathway activity in the fresh-frozen xenograft samples processed according to the Affymetrix One-Cycle protocol versus the MessageAmp Premier protocol. As shown in Figure 3B, the probability of RAS and MYC pathway activities correlates extremely well between a given pair of fresh-frozen xenograft samples processed according to the Affymetrix One-Cycle protocol versus the MessageAmp Premier protocol (RAS p < 0.0001, MYC p = 0.011).

**Figure 4:**
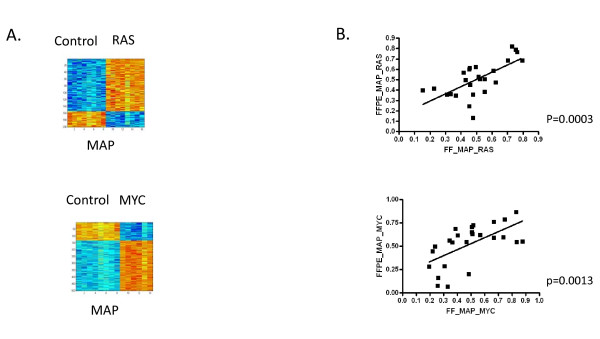
**Status of RAS and MYC pathway activities in paired fresh-frozen and FFPE xenografts**. (A) Heatmaps representing the expression levels of genes comprising the gene expression signature that differentiates between HMECs infected with adenovirus expressing GFP (control) versus HMECs infected with adenovirus expressing RAS or MYC as indicated. Within the heatmap, columns represent samples and rows represent genes, with expression values on a low to high continuum represented by a blue to red continuum, respectively. (B) Scatterplot of the fresh-frozen xenografts (X-axis) versus the FFPE xenografts (Y-axis) for the RAS pathway (top) or MYC pathway (bottom). The simple linear regression model and p-value is drawn for each Pearson correlation.

As shown in Figure 4, we predicted the activities of the RAS and MYC pathways in the fresh-frozen and FFPE xenograft samples to determine if consistent information about the status of oncogenic signaling pathway activities can be obtained from expression data from paired fresh-frozen and FFPE xenograft samples. As shown in Figure 4A, a predictor consisting of 200 genes or 500 genes differentiates between HMECs expressing RAS and MYC, respectively, relative to HMECs expressing GFP. These predictors were then used to predict the status of the RAS and MYC pathways in the fresh-frozen and FFPE xenograft samples processed according to the MessageAmp Premier protocol. As shown in Figure 4B, the predicted probability of RAS and MYC pathway activity correlates extremely well between a given pair of fresh-frozen and FFPE xenograft samples (RAS p = 0.0003, MYC p = 0.0013).

### Prediction of signaling pathways in patient matched fresh-frozen and formalin-fixed, paraffin-embedded melanoma samples

**Figure 5:**
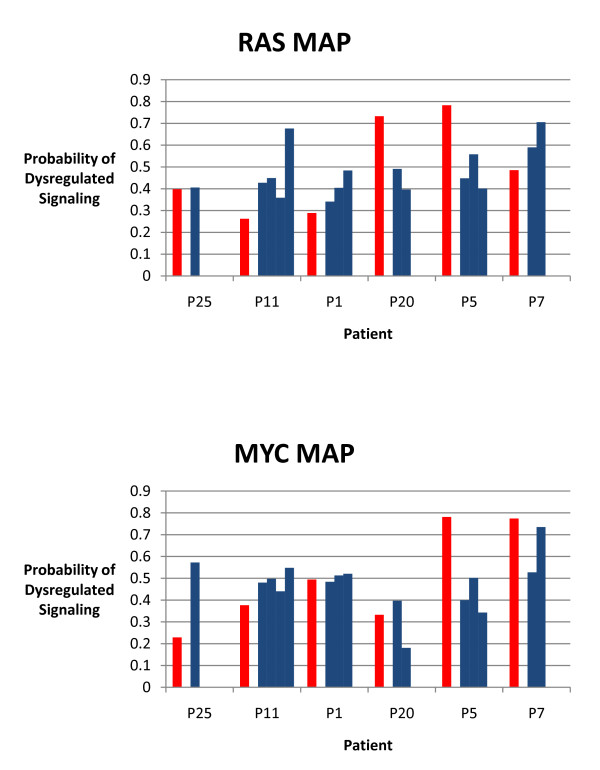
**Status of RAS and MYC pathway activities in patient matched fresh-frozen and FFPE melanomas**. Graphs represent the pathway predictions for the status of the RAS pathway (top) and MYC pathway (bottom) in fresh-frozen and FFPE samples for patients as indicated. Blue bars represent fresh-frozen samples and red bars represent FFPE samples.

Having demonstrated that the status of oncogenic signaling pathways can be consistently predicted using gene expression data obtained from paired fresh-frozen and FFPE xenograft samples, we investigated whether the status of oncogenic signaling pathways could also be consistently predicted using gene expression data obtained from patient matched fresh-frozen and FFPE melanomas. Fresh-frozen samples for these analyses consisted of in-transit melanomas from 6 patients obtained before the initiation of melphalan isolated limb infusion chemotherapy. Previous work has predicted the status of oncogenic signaling pathways in these samples and has shown that the status of a particular oncogenic signaling pathway is concordant across the lesions from a given patient, with the range of predictions not exceeding 0.3 in 70% or more of the patients [14]. FFPE tissue from a lesion from each patient within a month prior to the initiation of melphalan isolated limb infusion chemotherapy was obtained and the status of the RAS and MYC oncogenic signaling pathways were predicted. As shown in Figure 5, RAS pathway status showed a strong concordance (100%) in fresh-frozen and FFPE samples from a given patient, as the difference in the probability of RAS pathway activity in the FFPE samples and at least one of the fresh-frozen in-transit lesions did not exceed 0.3 for any of the patients. The average within-sample differences seen in patients was 0.184 (ranging from 0.007 to 0.314). MYC pathway status also showed a strong concordance (83%) in fresh-frozen and FFPE samples from a given patient, as the difference in the probability of MYC pathway activity in the FFPE samples and at least one of the fresh-frozen in-transit lesions did not exceed 0.3 for 5 of the 6 patients. The average within-sample differences seen in patients was 0.182 (ranging from 0.019 to 0.367).

## Conclusions

Genomic profiling has been shown to play an important role in characterizing distinct forms of cancers on the basis of patterns of gene expression and functions associated with genes relevant to profiles. These characterizations provide potential approaches to further understand the biology underlying individual tumors and to develop new targeted therapeutic options for subgroups of patients, matching targeted therapies in a rational way with characteristics of the patient's tumor. An ability to use FFPE tissue samples for gene expression profiling will facilitate the number and type of available samples for research analyses as well as allow these assays to be done in a clinical setting.

Prior studies have described the use of Quantitative Real-Time Polymerase Chain Reaction (QRT-PCR) assays as a method to measure gene expression within FFPE tumor tissue samples. While these assays can be informative with respect to measuring the expression of specific genes in a tumor sample, this approach does not provide a basis for whole genome expression measurement. As such, only a restricted view of the underlying biology of the tumor can be obtained and discovery of new genomic profiles relevant to the tumor cannot be generated. Thus, the capacity to employ whole-genome expression measurements from FFPE tumor tissue samples is critical.

The work we describe here demonstrates the development of an assay to enable genomic signatures that can measure deregulation of various oncogenic signaling pathways to be applied to FFPE tumor tissue samples. Although genome wide expression data correlates poorly between paired fresh-frozen and FFPE xenograft samples, it is apparent that consistent and reliable genomic profiles reflecting activities of oncogenic signaling pathways comprised of a subset of probes that exhibit a consistent pattern of expression associated with the expression of the activated oncogene can be generated. We believe this reflects the complexity of these cell signaling pathways combined with the power of the signature development approach whereby the capacity to sample a diverse array of expression values can yield measures of pathway activity. We have shown that RNA isolated from FFPE tumor tissue samples, amplified following the MessageAmp Premier protocol, and hybridized to Affymetrix arrays can be used to generate consistent and reliable genomic profiles reflecting RAS and MYC pathway activity. We have shown significant correlations between predictions of the status of the RAS and MYC pathways in paired fresh-frozen and FFPE xenograft samples. In addition, we have shown significant correlations between the pathway predictions in patient matched fresh-frozen and FFPE melanoma samples. In future work it will be critical to evaluate the ability of this assay to generate valid genomic predictions from prospectively collected FFPE samples in a clinical trial.

An ability to generate quality whole-genome expression data from FFPE tumor tissue samples allows the large number of currently banked samples to be used in genomic profiling analyses. This not only gives researchers access to a tremendous number of samples, but also allows researchers to utilize samples from patients for which clinical outcomes are known. Furthermore, the ability to generate genomic profiles using this assay from prospectively collected FFPE tumor tissue samples in clinical trials has the potential to enable clinicians to utilize the information of the underlying biology of tumors from individual patients to more accurately diagnose an individual patient's disease, more properly predict the course of an individual patient's disease, and more rationally match therapeutic options with an individual patient's disease.

## Competing interests

Dr. Douglas Tyler is a recipient of commercial research grants from Scherring-Plough and Adherex. He has received an honorarium from the Novartis speaker's bureau. He is a member of the Genetech Scientific Advisory Board. Dr. Joseph Nevins has ownership interest in Expression Analysis. He is a member of the Millenium Pharmaceuticals and Qiagen Scientific Advisory Boards.

## Authors' contributions

JAF participated in conceiving the study, carrying out the study, and drafting the manuscript. CKA participated in conceiving the study, generated the xenografts, provided the human melanoma samples, and participated in drafting the manuscript. MAS participated in procurement of the human melanoma samples. KCH carried out the RNA amplifications and hybridizations to the GeneChips. ZW participated in the statistical analyses. KAT participated in carrying out the RNA preparations. DSH participated in conceiving the study. HKD participated in conceiving the study. WTB participated in the statistical analyses and in drafting the manuscript. DST participated in conceiving the study and provided the xenografts and human melanoma samples. JRN participated in conceiving the study and in drafting the manuscript. All authors read and approved the final manuscript.

## Pre-publication history

The pre-publication history for this paper can be accessed here:

http://www.biomedcentral.com/1755-8794/4/58/prepub

## Supplementary Material

Additional file 1 **Supplementary methods**. A supplementary methods file including greater detail of pathway analyses. Click here for file

Additional file 2 **Principal component analyses of the MAS5.0 normalized gene expression data from the fresh-frozen xenografts processed according to the Affymetrix One-Cycle protocol versus the MessageAmp Premier protocol**. (A) Principal component analyses in greater than 2 dimensions. Shapes represent samples and colors represent the processing protocol used, with all samples processed according to the Affymetrix One-Cycle protocol depicted in black and all samples processed according to the MessageAmp Premier protocol depicted in red. (B) Graph depicting the percentage of variance that each principal component captures. Click here for file

Additional file 3 **Heat map representing the correlation coefficients for whole-genome expression data obtained from fresh-frozen xenografts processed according to the Affymetrix One-Cycle protocol versus the MessageAmp Premier protocol**. Within the heat map, columns represent fresh-frozen xenografts processed according to the MessageAmp Premier protocol and rows represent fresh-frozen xenografts processed according to the Affymetrix One-Cycle protocol. Correlation coefficients represented on a yellow to red continuum, as indicated. Click here for file

Additional file 4 **Principal component analyses of the MAS5.0 normalized gene expression data from the fresh-frozen xenografts versus the FFPE xenografts processed according to the MessageAmp Premier protocol**. (A) Principal component analyses in greater than 2 dimensions. Given pairs of fresh-frozen and FFPE samples are depicted by the same shape with fresh-frozen samples depicted in black and FFPE samples depicted in red. (B) Graph depicting the variance that each principal component captures. Click here for file

Additional file 5 **Heat map representing the correlation coefficients for whole-genome expression data obtained from fresh-frozen xenografts versus FFPE xenografts processed according to the MessageAmp Premier protocol**. Within the heat map, columns represent fresh-frozen xenografts and rows represent FFPE xenografts. Correlation coefficients represented on a yellow to red continuum, as indicated. Click here for file

Additional file 6 **Probes comprising signatures**. A file including the probes comprising each signature. Click here for file
