# Supplementary material for: A methodology for utilization of predictive genomic signatures in FFPE samples
Source: BMC Med Genomics. 2011 Jul 11;4:58. doi: 10.1186/1755-8794-4-58 (PMC3146808; doi:10.1186/1755-8794-4-58)
Supplement: Additional file 1 — Supplementary methods. A supplementary methods file including greater detail of pathway analyses. [file 1755-8794-4-58-S1.DOCX]

**Supplementary Methods**

Analyses were performed using a Dell desktop computer with a 2.8GHz Intel Pentium D CPU processor, 2 Gb RAM, and running Microsoft Windows XP Professional 2002. All analyses, unless otherwise stated, were performed using MATLAB with the Bioinformatics and Statistical toolboxes installed. Probes comprising all signatures are listed in the Probes_comprising_signatures_BMC_Medical_Genomics.pdf file.

**Generation of RAS and MYC pathway predictions using fresh-frozen xenograft samples processed according to the Affymetrix One-Cycle protocol versus the MessageAmp Premier protocol.**

Using the normalize.R script (available at http://data.genome.duke.edu/Freedman_CEL_Files) run in R (ver2.6.0), .CEL files were RMA normalized. The methods used to generate the oncogene and tumor suppressor pathway signatures have been described in great detail by Gatza *et al*. 2010 [1]. MATLAB release R2008a was used for the analyses. The Binreg ver2 program and tutorial is available at <http://www.duke.edu/~dinbarry/BINREG/>.

The signatures (RAS/Affymetrix One-Cycle built using data for g2, g3, g4, g5, g6, g7, g8, g9, r1, r2, r3, r5, r6, r7, r8, r9 samples processed according to the Affymetrix One-Cycle protocol, RAS/MessageAmp Premier built using data for g2, g3, g4, g5, g6, g7, g8, g9, r1, r2, r3, r5, r6, r7, r8, r9 samples processed according to the MessageAmp Premier protocol, MYC/Affymetrix One-Cycle built using data for g2, g3, g4, g5, g6, g7, g8, g9, m3, m4, m5, m6, m7, m8 samples processed according to the Affymetrix One-Cycle protocol, MYC/MessageAmp Premier built using data for g2, g3, g4, g5, g7, g8, g9, m3, m4, m5, m6, m7, m8 samples processed according to the MessageAmp Premier protocol) were applied to the fresh-frozen xenograft samples processed according to the Affymetrix One-Cycle protocol versus the MessageAmp Premier protocol using the following parameters:

| Signature | MYC | RAS |
| --- | --- | --- |
| Genes | 500 | 350 |
| Metagenes | 2 | 2 |
| Burn-in | 1000 | 1000 |
| Iterations | 5000 | 5000 |
| Skips | 1 | 1 |
| CI | 95% | 95% |
| RMA/MAS5 | RMA | RMA |
| Quantile Normalize | No | No |
| Shift scale | Yes | Yes |
| Binreg Version | 2 | 2 |

**Generation of RAS and MYC pathway predictions using fresh-frozen versus FFPE xenograft samples processed according to the MessageAmp Premier protocol.**

Using Affymetrix Expression Console (ver1.1), .CEL files were MAS5 normalized. The methods used to generate the oncogene and tumor suppressor pathway signatures have been described in great detail by Gatza *et al*. 2010 [1]. MATLAB release R2008a was used for the analyses. The Binreg ver2 program and tutorial is available at <http://www.duke.edu/~dinbarry/BINREG/>.

The signatures (RAS/MessageAmp Premier built using data for g2, g3, g4, g5, g6, g7, g8, g9, r1, r2, r3, r5, r6, r7, r8, r9 samples processed according to the MessageAmp Premier protocol, MYC/MessageAmp Premier built using data for g2, g3, g4, g5, g6, g7, g8, g9, m3, m4, m5, m6, m7, m8 samples processed according to the MessageAmp Premier protocol) were applied to the fresh-frozen and FFPE xenograft samples processed according to the MessageAmp Premier protocol using the following parameters:

| Signature | MYC | RAS |
| --- | --- | --- |
| Genes | 500 | 200 |
| Metagenes | 2 | 1 |
| Burn-in | 1000 | 1000 |
| Iterations | 5000 | 5000 |
| Skips | 1 | 1 |
| CI | 95% | 95% |
| RMA/MAS5 | MAS5 | MAS5 |
| Quantile Normalize | Yes | Yes |
| Shift scale | Yes | Yes |
| Binreg Version | 2 | 2 |

**Generation of RAS and MYC pathway predictions using FFPE human melanoma samples processed according to the MessageAmp Premier protocol.**

Using Affymetrix Expression Console (ver1.1), .CEL files were MAS5 normalized. The methods used to generate the oncogene and tumor suppressor pathway signatures have been described in great detail by Gatza *et al*. 2010 [1]. MATLAB release R2008a was used for the analyses. The Binreg ver2 program and tutorial is available at <http://www.duke.edu/~dinbarry/BINREG/>. The signatures were applied to the FFPE human melanoma samples processed according to the MessageAmp Premier protocol using the same parameters as indicated above for the generation of RAS and MYC pathway predictions using fresh-frozen versus FFPE xenograft samples processed according to the MessageAmp Premier protocol.

**References**

1. Gatza ML, Lucas JE, Barry WT, Kim J-W, Wang Q, Crawford M, Datto M, Kelley M, Mathey-Prevot B, Potti A, Nevins JR: **A pathway-based classification of human breast cancer**. *Proc Nat'l Acad Sci* 2010, **107**:6994-6999.
