# Supplementary figures and images for: A methodology for utilization of predictive genomic signatures in FFPE samples
Source: BMC Med Genomics. 2011 Jul 11;4:58. doi: 10.1186/1755-8794-4-58 (PMC3146808; doi:10.1186/1755-8794-4-58)

A.

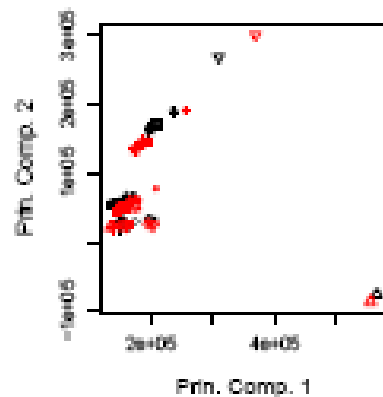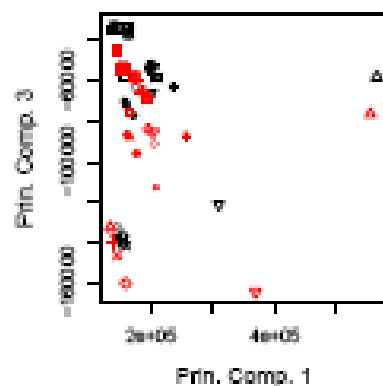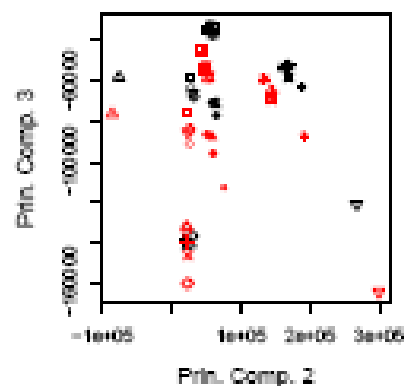

B.

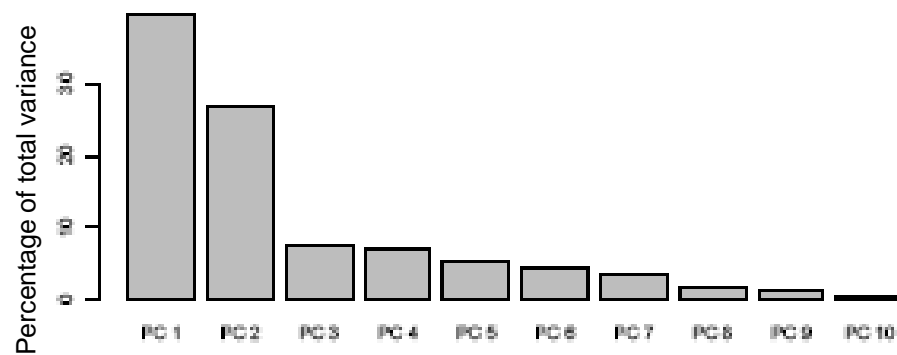

Supplement: Additional file 2 — Principal component analyses of the MAS5.0 normalized gene expression data from the fresh-frozen xenografts processed according to the Affymetrix One-Cycle protocol versus the MessageAmp Premier protocol. (A) Principal component analyses in greater than 2 dimensions. Shapes represent samples and colors represent the processing protocol used, with all samples processed according to the Affymetrix One-Cycle protocol depicted in black and all samples processed according to the MessageAmp Premier protocol depicted in red. (B) Graph depicting the percentage of variance that each principal component captures. [file 1755-8794-4-58-S2.PDF]

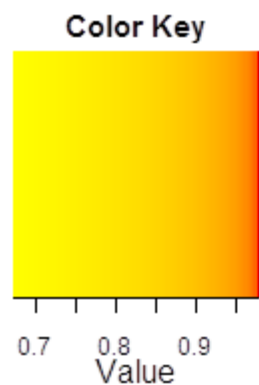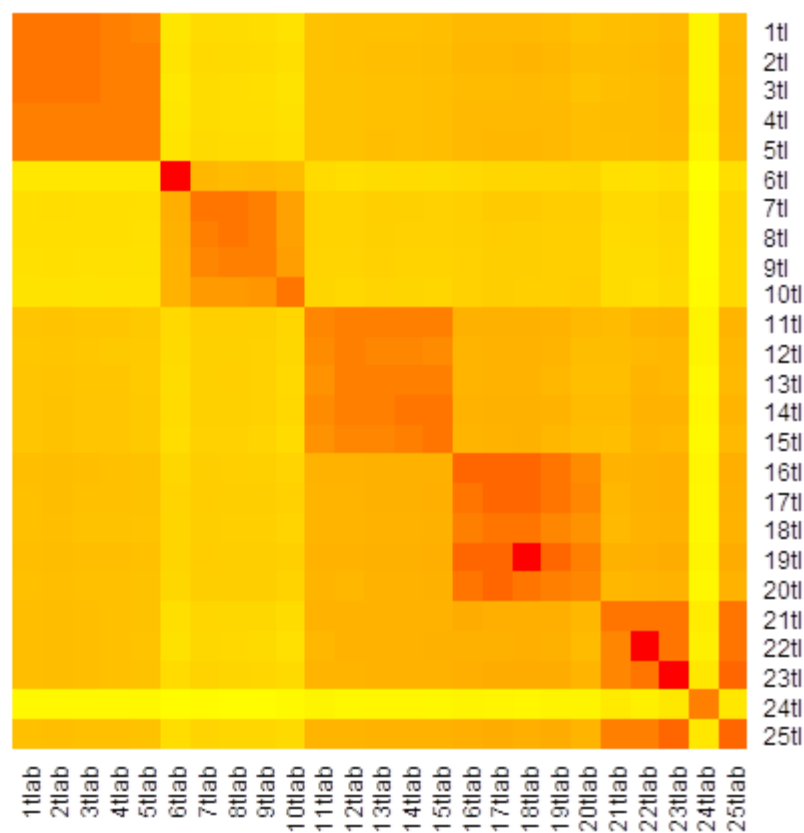

Affymetrix One-Cycle processed

MessageAmp Premier processed

Supplement: Additional file 3 — Heat map representing the correlation coefficients for whole-genome expression data obtained from fresh-frozen xenografts processed according to the Affymetrix One-Cycle protocol versus the MessageAmp Premier protocol. Within the heat map, columns represent fresh-frozen xenografts processed according to the MessageAmp Premier protocol and rows represent fresh-frozen xenografts processed according to the Affymetrix One-Cycle protocol. Correlation coefficients represented on a yellow to red continuum, as indicated. [file 1755-8794-4-58-S3.PDF]

A.

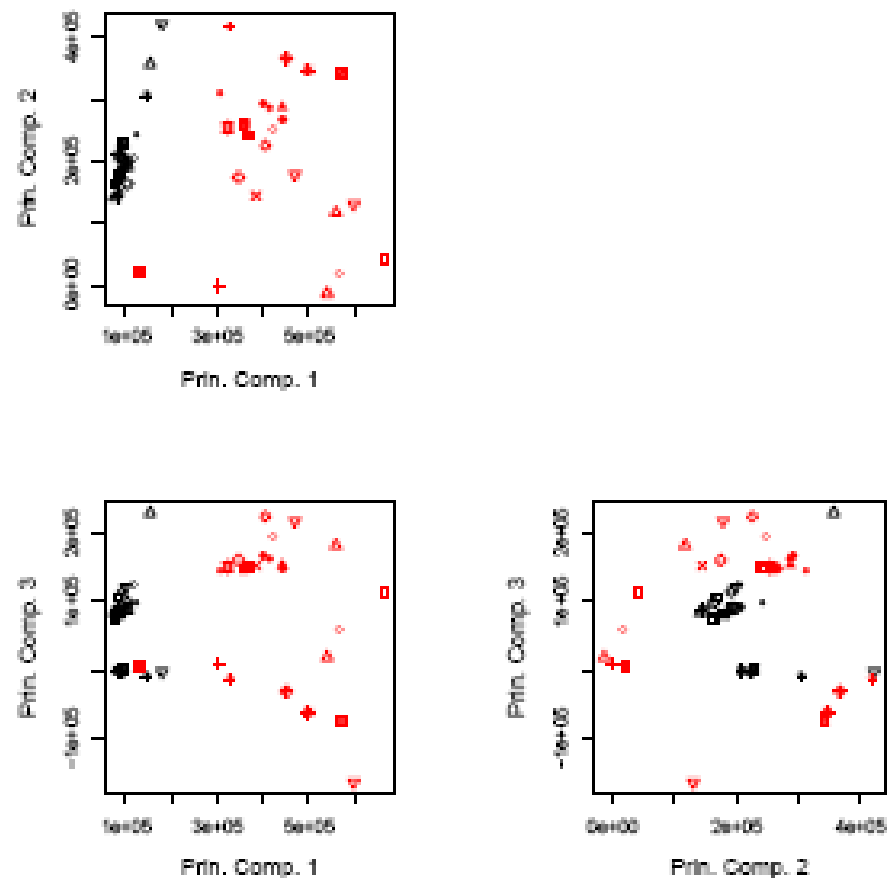

B.

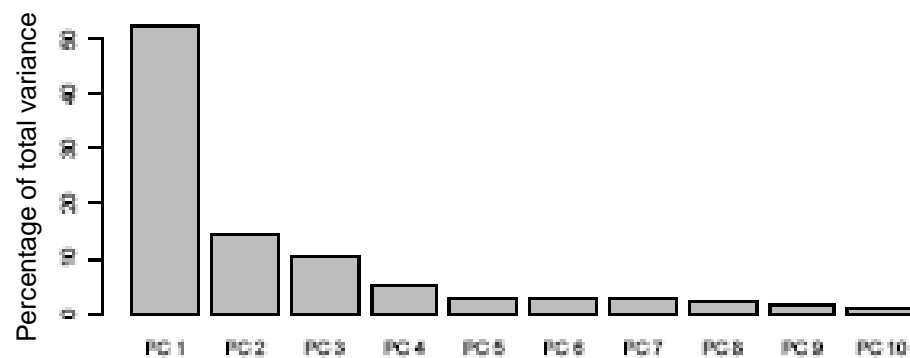

Supplement: Additional file 4 — Principal component analyses of the MAS5.0 normalized gene expression data from the fresh-frozen xenografts versus the FFPE xenografts processed according to the MessageAmp Premier protocol. (A) Principal component analyses in greater than 2 dimensions. Given pairs of fresh-frozen and FFPE samples are depicted by the same shape with fresh-frozen samples depicted in black and FFPE samples depicted in red. (B) Graph depicting the variance that each principal component captures. [file 1755-8794-4-58-S4.PDF]

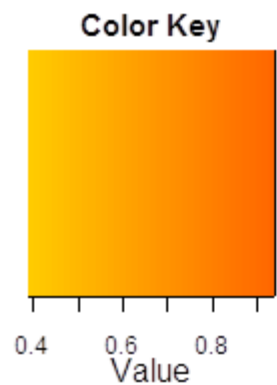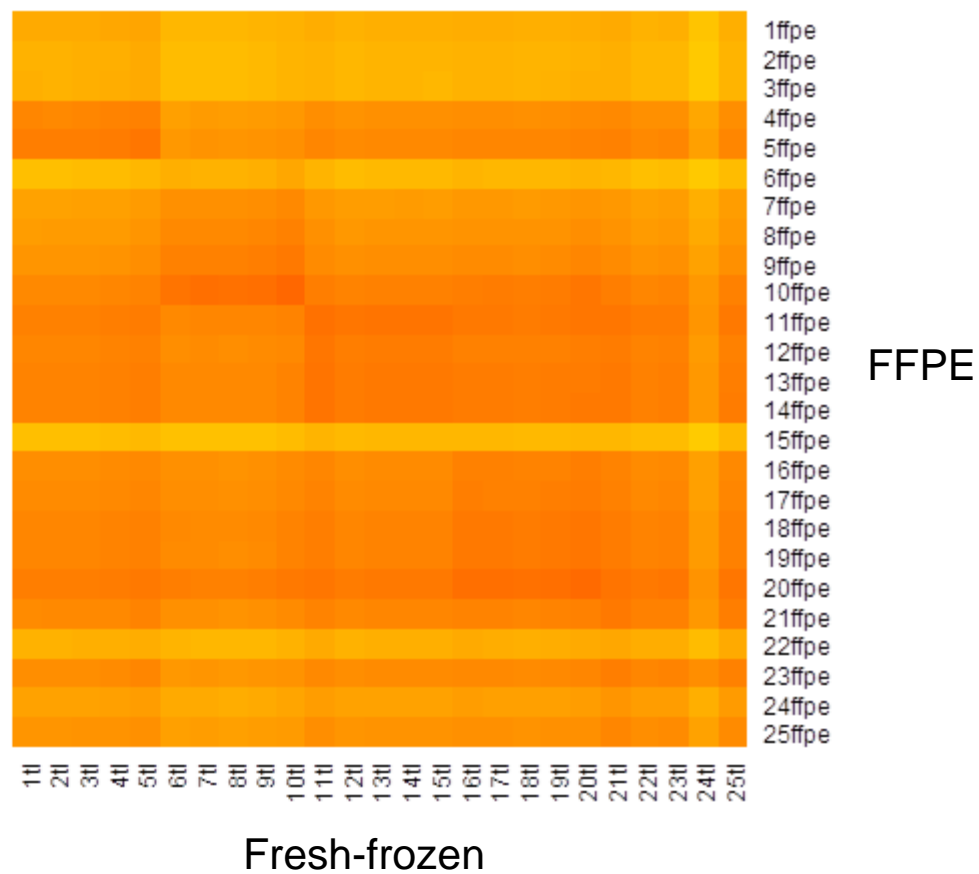

Supplement: Additional file 5 — Heat map representing the correlation coefficients for whole-genome expression data obtained from fresh-frozen xenografts versus FFPE xenografts processed according to the MessageAmp Premier protocol. Within the heat map, columns represent fresh-frozen xenografts and rows represent FFPE xenografts. Correlation coefficients represented on a yellow to red continuum, as indicated. [file 1755-8794-4-58-S5.PDF]
