# Supplementary material for: A methodology for utilization of predictive genomic signatures in FFPE samples
Source: BMC Med Genomics. 2011 Jul 11;4:58. doi: 10.1186/1755-8794-4-58 (PMC3146808; doi:10.1186/1755-8794-4-58)
Supplement: Additional file 6 — Probes comprising signatures. A file including the probes comprising each signature. [file 1755-8794-4-58-S6.PDF]

RAS_One_Cycle (Fig 3)
222227_at
205034_at
211814_s_at
202860_at
204768_s_at
208893_s_at
201555_at
217996_at
204015_s_at
201473_at
39402_at
205476_at
202086_at
216237_s_at
204745_x_at
201631_s_at
209189_at
204439_at
218719_s_at
212188_at
202081_at
208892_s_at
208891_at
41469_at
205067_at
201930_at
214022_s_at
212192_at
214453_s_at
38149_at
211058_x_at
217997_at
209122_at
204794_at
219836_at
204995_at
205687_at
202107_s_at
201695_s_at
221489_s_at
205909_at
202174_s_at
206631_at
206127_at
200797_s_at
201041_s_at

209039_x_at
204803_s_at
60474_at
209457_at
209773_s_at
211756_at
205289_at
201564_s_at
201939_at
201642_at
207850_at
202503_s_at
203968_s_at
212185_x_at
217998_at
218951_s_at
218782_s_at
214391_x_at
213088_s_at
59705_at
211924_s_at
203821_at
222037_at
201042_at
212983_at
204470_at
204359_at
220081_x_at
205479_s_at
204802_at
212282_at
206461_x_at
209366_x_at
209482_at
201890_at
218585_s_at
211750_x_at
219258_at
205024_s_at
210189_at
216189_at
210845_s_at
204614_at
203992_s_at
205680_at
201218_at
211450_s_at

209278_s_at
214059_at
209943_at
201277_s_at
220651_s_at
203612_at
218751_s_at
204715_at
211576_s_at
201470_at
206176_at
212434_at
222036_s_at
210001_s_at
212320_at
218796_at
202693_s_at
206300_s_at
209567_at
203696_s_at
201090_x_at
203209_at
216875_x_at
212141_at
208977_x_at
217165_x_at
212253_x_at
211072_x_at
202911_at
206115_at
213726_x_at
205767_at
206052_s_at
210346_s_at
201092_at
220675_s_at
214011_s_at
205393_s_at
201170_s_at
213603_s_at
218997_at
203882_at
210387_at
208114_s_at
209803_s_at
204023_at
58916_at

219060_at
202912_at
209375_at
217603_at
217858_s_at
208730_x_at
203217_s_at
201202_at
222162_s_at
218350_s_at
215310_at
220615_s_at
221521_s_at
38037_at
206109_at
204012_s_at
213293_s_at
203967_at
215012_at
212639_x_at
213851_at
201464_x_at
213646_x_at
222303_at
205053_at
221009_s_at
218862_at
206526_at
201512_s_at
211668_s_at
202346_at
209958_s_at
219006_at
214240_at
202695_s_at
204695_at
201376_s_at
213523_at
204514_at
218224_at
218247_s_at
208581_x_at
205229_s_at
219105_x_at
212859_x_at
210126_at
204011_at

207419_s_at
205272_s_at
212977_at
212268_at
212281_s_at
211506_s_at
212754_s_at
202870_s_at
200644_at
204767_s_at
202310_s_at
203210_s_at
216336_x_at
204882_at
204475_at
204146_at
209421_at
208937_s_at
215659_at
205016_at
213951_s_at
205345_at
213524_s_at
212432_at
219403_s_at
200790_at
218069_at
213132_s_at
214408_s_at
210268_at
209447_at
201328_at
201700_at
200773_x_at
217995_at
204612_at
210452_x_at
213708_s_at
208076_at
205676_at
220688_s_at
202241_at
219177_at
213292_s_at
221773_at
200799_at
201489_at

212168_at
209251_x_at
219419_at
218346_s_at
203432_at
1053_at
204440_at
212455_at
208795_s_at
202431_s_at
200650_s_at
204855_at
212330_at
201889_at
202655_at
179_at
222221_x_at
211456_x_at
200911_s_at
201781_s_at
205021_s_at
202437_s_at
208816_x_at
202435_s_at
200875_s_at
214683_s_at
212658_at
207339_s_at
209037_s_at
201152_s_at
204510_at
208642_s_at
221840_at
205290_s_at
218971_s_at
209697_at
204026_s_at
214734_at
204014_at
213610_s_at
203664_s_at
205394_at
215629_s_at
202132_at
201860_s_at
208308_s_at
218866_s_at

204028_s_at
207843_x_at
204798_at
203418_at
204247_s_at
211573_x_at
201563_at
209598_at
201628_s_at
219648_at
205895_s_at
217369_at
209158_s_at
200610_s_at
204610_s_at
219179_at
218461_at
209679_s_at
206034_at
218092_s_at
211725_s_at
218647_s_at
218708_at
209478_at
219269_at
206088_at
203094_at
207655_s_at
205266_at
218605_at
217164_at
205490_x_at
213629_x_at
207844_at
210118_s_at
219545_at
217980_s_at
218644_at
209718_at
215678_at
210559_s_at
219494_at
214710_s_at
217614_at
220079_s_at
203002_at
204286_s_at

211386_at
204886_at
204326_x_at
218738_s_at
208758_at
212254_s_at
202133_at
201325_s_at
217696_at
202330_s_at
203348_s_at
205249_at
213911_s_at
221978_at
208766_s_at
206156_at
218001_at
202138_x_at
204420_at
204268_at
220945_x_at
201947_s_at

RAS_MAP (Fig 3)
222227_at
205034_at
201466_s_at
60474_at
208893_s_at
205676_at
203882_at
202241_at
212185_x_at
208891_at
215243_s_at
203821_at
211448_s_at
200037_s_at
203391_at
202912_at
214760_at
204326_x_at
201042_at
38037_at
218751_s_at
209447_at
207655_s_at

204470_at
203002_at
204072_s_at
206461_x_at
208892_s_at
217997_at
217996_at
202230_s_at
201041_s_at
218277_s_at
205067_at
218796_at
209189_at
209803_s_at
215111_s_at
208961_s_at
204015_s_at
211756_at
205878_at
201563_at
206176_at
221489_s_at
219255_x_at
214453_s_at
204011_at
204614_at
218513_at
215645_at
202414_at
201473_at
207850_at
208152_s_at
221493_at
212281_s_at
202860_at
201930_at
220945_x_at
202081_at
218247_s_at
205068_s_at
220317_at
206156_at
222162_s_at
36711_at
209251_x_at
203894_at
208598_s_at

202911_at
218878_s_at
208450_at
208766_s_at
204995_at
212320_at
214093_s_at
205084_at
201090_x_at
218440_at
208712_at
212192_at
219161_s_at
215412_x_at
221485_at
203931_s_at
207506_at
201202_at
219494_at
215988_s_at
212745_s_at
218826_at
203418_at
202936_s_at
213080_x_at
201695_s_at
203044_at
209184_s_at
216821_at
179_at
200603_at
207434_s_at
205289_at
221567_at
213848_at
201324_at
201009_s_at
202107_s_at
204462_s_at
206688_s_at
201631_s_at
219526_at
209476_at
209604_s_at
216189_at
204182_s_at
212188_at

201177_s_at
213358_at
221961_at
212458_at
212330_at
209457_at
216525_x_at
204803_s_at
214451_at
214753_at
204475_at
203139_at
210983_s_at
206052_s_at
212536_at
209598_at
203696_s_at
218871_x_at
208503_s_at
203203_s_at
215990_s_at
213372_at
201641_at
200847_s_at
205227_at
202435_s_at
201555_at
204526_s_at
210346_s_at
205476_at
219836_at
205306_x_at
39582_at
205490_x_at
219893_at
209544_at
218914_at
203567_s_at
212551_at
204510_at
218997_at
204181_s_at
205016_at
218585_s_at
204420_at
220079_s_at
205642_at

214972_at
212752_at
203312_x_at
219105_x_at
221195_at
213236_at
202458_at
209211_at
205263_at
204026_s_at
206115_at
212390_at
202330_s_at
212455_at
205290_s_at
204126_s_at
201013_s_at
218644_at
34408_at
218460_at
201694_s_at
207566_at
218971_s_at
208841_s_at
221660_at
214059_at
222303_at
221521_s_at
206114_at
207719_x_at
208114_s_at
204855_at
202666_s_at
204359_at
209433_s_at
218358_at
211814_s_at
209375_at
213486_at
205266_at
205767_at
221887_s_at
221823_at
200919_at
200747_s_at
209118_s_at
218712_at

218987_at
201479_at
204882_at
202314_at
211456_x_at
204146_at
201446_s_at
221730_at
209122_at
202436_s_at
208711_s_at
215191_at
212254_s_at
202859_x_at
212282_at
217509_x_at
220651_s_at
213438_at
213646_x_at
204948_s_at
221773_at
200773_x_at
217168_s_at
218738_s_at
212253_x_at
203325_s_at
205190_at
202503_s_at
205622_at
208816_x_at
218397_at
213311_s_at
202934_at
201328_at
213546_at
214925_s_at
210845_s_at
221510_s_at
222037_at
221009_s_at
212660_at
210707_x_at
214240_at
210852_s_at
212639_x_at
205085_at
215228_at

203401_at
214475_x_at
214755_at
201294_s_at
213295_at
204028_s_at
204268_at
203177_x_at
205194_at
222344_at
220482_s_at
218346_s_at
202126_at
211072_x_at
221558_s_at
201890_at
205619_s_at
38149_at
202433_at
216515_x_at
219006_at
205909_at
205483_s_at
39402_at
210944_s_at
201407_s_at
221774_x_at
219269_at
200644_at
204745_x_at
220148_at
218602_s_at
208795_s_at
212870_at
217165_x_at
218579_s_at
211951_at
208475_at
204415_at
205327_s_at
205479_s_at
210265_x_at
206020_at
206363_at
221011_s_at
204695_at
217976_s_at

202393_s_at
208474_at
212766_s_at
202967_at
200790_at
37152_at
212994_at
220675_s_at
209124_at
205828_at
211058_x_at
203518_at
201693_s_at
201354_s_at
204669_s_at
215096_s_at
204802_at
203315_at
204439_at
211764_s_at
206300_s_at
219545_at
202613_at
202086_at
212704_at
209278_s_at
214946_x_at
213523_at
217373_x_at
200594_x_at
220623_s_at
203674_at
210396_s_at
216350_s_at
208581_x_at
215016_x_at
213435_at
214011_s_at
211750_x_at
206569_at
201503_at
201092_at
214132_at
217739_s_at
205249_at

MYC_One_Cycle (Fig 3)

222227_at
202431_s_at
209433_s_at
200873_s_at
201391_at
201695_s_at
210347_s_at
212846_at
202364_at
221586_s_at
207515_s_at
204794_at
208758_at
201872_s_at
217858_s_at
218889_at
218882_s_at
204027_s_at
200875_s_at
216212_s_at
214813_at
212922_s_at
218069_at
212510_at
218590_at
222305_at
218398_at
212281_s_at
203882_at
220607_x_at
211576_s_at
201512_s_at
216913_s_at
202078_at
201614_s_at
210625_s_at
219131_at
218156_s_at
208778_s_at
204602_at
221575_at
220865_s_at
204748_at
219037_at
218278_at
219244_s_at
209189_at

213132_s_at
211951_at
208038_at
635_s_at
222013_x_at
205330_at
208985_s_at
221025_x_at
216705_s_at
209366_x_at
219006_at
215009_s_at
212635_at
201278_at
214734_at
208795_s_at
209196_at
218168_s_at
215031_x_at
214794_at
218594_at
219178_at
212333_at
220212_s_at
201420_s_at
208822_s_at
208644_at
217956_s_at
40446_at
208117_s_at
207396_s_at
202309_at
201608_s_at
218605_at
218959_at
211954_s_at
200658_s_at
219419_at
210466_s_at
203150_at
203939_at
201562_s_at
221677_s_at
221020_s_at
219217_at
209776_s_at
218027_at

201873_s_at
208308_s_at
203612_at
217848_s_at
202776_at
218561_s_at
214453_s_at
218997_at
200773_x_at
218592_s_at
219715_s_at
210988_s_at
205895_s_at
205774_at
203610_s_at
218512_at
204493_at
213189_at
220789_s_at
217832_at
220688_s_at
219969_at
220709_at
205264_at
221987_s_at
212411_at
201555_at
206133_at
218239_s_at
209317_at
204012_s_at
206554_x_at
211623_s_at
219111_s_at
201478_s_at
201013_s_at
203023_at
212145_at
209943_at
212378_at
205306_x_at
214011_s_at
205113_at
204547_at
217099_s_at
210921_at
200650_s_at

209567_at
213235_at
210027_s_at
212910_at
201014_s_at
200903_s_at
217850_at
204514_at
214193_s_at
211725_s_at
214391_x_at
204331_s_at
205942_s_at
218858_at
206631_at
212904_at
201491_at
204700_x_at
203148_s_at
210757_x_at
200874_s_at
201325_s_at
65585_at
214661_s_at
219031_s_at
221036_s_at
218724_s_at
203108_at
217724_at
204033_at
219198_at
218862_at
219522_at
202973_x_at
212282_at
205135_s_at
215165_x_at
203867_s_at
209864_at
200050_at
212885_at
209233_at
218088_s_at
212541_at
218529_at
204133_at
205527_s_at

204610_s_at
205249_at
202529_at
212192_at
203119_at
215046_at
212175_s_at
214283_at
213293_s_at
204141_at
210076_x_at
217809_at
218270_at
217369_at
219539_at
209434_s_at
219345_at
218408_at
211762_s_at
217987_at
212018_s_at
209731_at
204521_at
216262_s_at
203360_s_at
212379_at
203567_s_at
220060_s_at
213627_at
201112_s_at
201323_at
208264_s_at
208114_s_at
215438_x_at
211163_s_at
209814_at
205217_at
203405_at
200063_s_at
213320_at
204803_s_at
200610_s_at
219575_s_at
204215_at
215091_s_at
208766_s_at
219884_at

202159_at
201266_at
203362_s_at
217791_s_at
212114_at
219147_s_at
204175_at
200726_at
204359_at
65588_at
221168_at
204405_x_at
207610_s_at
218897_at
201327_s_at
203622_s_at
204542_at
200662_s_at
218316_at
210008_s_at
202268_s_at
212168_at
200828_s_at
209725_at
218866_s_at
200993_at
219494_at
209824_s_at
219581_at
212563_at
213418_at
203518_at
218479_s_at
208642_s_at
201489_at
219498_s_at
41037_at
205963_s_at
221786_at
218710_at
212658_at
201892_s_at
218888_s_at
218073_s_at
211949_s_at
200910_at
218544_s_at

208828_at
203040_s_at
203910_at
212432_at
208436_s_at
209325_s_at
218199_s_at
217106_x_at
207843_x_at
221823_at
222360_at
59705_at
219716_at
201606_s_at
218647_s_at
207618_s_at
204905_s_at
217942_at
201912_s_at
202437_s_at
217754_at
179_at
215678_at
217792_at
216980_s_at
117_at
203051_at
206261_at
221691_x_at
214605_x_at
210840_s_at
201279_s_at
221712_s_at
202042_at
202690_s_at
201948_at
200005_at
210561_s_at
218481_at
222159_at
202801_at
204699_s_at
216515_x_at
33767_at
218365_s_at
201177_s_at
201076_at

204807_at
222064_s_at
218331_s_at
214484_s_at
200807_s_at
218595_s_at
201563_at
209153_s_at
218712_at
209171_at
40255_at
213333_at
201692_at
212558_at
205677_s_at
221514_at
201516_at
203568_s_at
205134_s_at
218001_at
217725_x_at
218984_at
211686_s_at
206277_at
201675_at
203721_s_at
209626_s_at
212038_s_at
221903_s_at
201376_s_at
218607_s_at
215380_s_at
216305_s_at
205565_s_at
219849_at
209478_at
209623_at
214507_s_at
219110_at
205115_s_at
201870_at
204802_at
214214_s_at
213929_at
218732_at
204135_at
202138_x_at

218708_at
201338_x_at
218305_at
200659_s_at
208897_s_at
200995_at
218461_at
214729_at
220773_s_at
220147_s_at
218096_at
209247_s_at
200037_s_at
209143_s_at
209181_s_at
217980_s_at
203234_at
209447_at
208787_at
212754_s_at
217834_s_at
218493_at
221688_s_at
220086_at
201433_s_at
205429_s_at
217047_s_at
214059_at
219295_s_at
221942_s_at
213226_at
213145_at
205004_at
209565_at
200687_s_at
209336_at
212841_s_at
213427_at
204977_at
214173_x_at
203712_at
202655_at
220647_s_at
212330_at
211097_s_at
214472_at
213573_at

212434_at
201115_at
213097_s_at
203277_at
218715_at
201030_x_at
218653_at
221941_at
201900_s_at
221978_at
203287_at
209971_x_at
219855_at
202706_s_at
221516_s_at
211953_s_at
202086_at
218970_s_at
217786_at
209575_at
212279_at
209406_at
213701_at
216685_s_at
204347_at
202246_s_at
214696_at
217595_at
214045_at
209332_s_at
201930_at
219439_at
209825_s_at
218976_at
212228_s_at
218507_at
221276_s_at
202346_at
218460_at
200627_at
212871_at
214753_at
201277_s_at
203341_at
207939_x_at
202522_at
213564_x_at

221923_s_at
202212_at
202613_at
201421_s_at
208696_at
221513_s_at
221649_s_at
219363_s_at
216397_s_at
201479_at
203648_at
204329_s_at
219177_at
221443_x_at
218238_at
212502_at
204244_s_at
221761_at
209161_at
219420_s_at
213608_s_at
207735_at
205210_at
1487_at
202972_s_at
201947_s_at
209100_at
210250_x_at
218670_at
205133_s_at

MYC_MAP (Fig 3)
222227_at
202431_s_at
210766_s_at
212218_s_at
201563_at
219537_x_at
218859_s_at
212455_at
218882_s_at
201503_at
209143_s_at
209567_at
209433_s_at
203149_at
218590_at

211951_at
208152_s_at
216212_s_at
212174_at
218427_at
202483_s_at
212145_at
218984_at
40446_at
201014_s_at
204514_at
201479_at
219098_at
205895_s_at
209366_x_at
205264_at
208758_at
201013_s_at
221277_s_at
218605_at
209623_at
209375_at
208114_s_at
211686_s_at
218595_s_at
203875_at
200992_at
209447_at
204977_at
202613_at
217884_at
200773_x_at
220311_at
201266_at
220212_s_at
40189_at
217724_at
212717_at
218001_at
204602_at
203712_at
214011_s_at
220688_s_at
203023_at
200988_s_at
212282_at
202144_s_at

204521_at
218653_at
201555_at
218982_s_at
211787_s_at
201516_at
204593_s_at
204905_s_at
203201_at
201074_at
200687_s_at
212173_at
218947_s_at
205129_at
205726_at
218592_s_at
212411_at
218481_at
221688_s_at
218888_s_at
209324_s_at
212281_s_at
203150_at
201491_at
219731_at
221970_s_at
203196_at
219539_at
221586_s_at
218886_at
215091_s_at
214059_at
220647_s_at
206376_at
209434_s_at
210616_s_at
200063_s_at
205565_s_at
200028_s_at
220933_s_at
201819_at
218866_s_at
206445_s_at
212846_at
203329_at
218981_at
221516_s_at

219110_at
205748_s_at
203200_s_at
201030_x_at
214214_s_at
202364_at
218512_at
212572_at
201128_s_at
214683_s_at
213132_s_at
204169_at
221020_s_at
210514_x_at
202690_s_at
222305_at
204547_at
201280_s_at
220547_s_at
201115_at
218239_s_at
221712_s_at
217509_x_at
218377_s_at
212254_s_at
210976_s_at
205774_at
201595_s_at
201478_s_at
205963_s_at
218594_at
218556_at
221691_x_at
210027_s_at
212137_at
219884_at
218069_at
205811_at
209971_x_at
201675_at
203518_at
210231_x_at
212434_at
216913_s_at
200910_at
200610_s_at
205527_s_at

201694_s_at
212558_at
204175_at
203931_s_at
215612_at
203119_at
220417_s_at
218997_at
215096_s_at
200875_s_at
208910_s_at
213188_s_at
213427_at
201326_at
201695_s_at
207507_s_at
218976_at
202928_s_at
205190_at
218238_at
201389_at
203944_x_at
213589_s_at
202963_at
221774_x_at
201692_at
218575_at
212922_s_at
212510_at
218893_at
201873_s_at
200005_at
218156_s_at
65588_at
219178_at
217833_at
214240_at
212992_at
209323_at
202973_x_at
214427_at
205340_at
205284_at
160020_at
218460_at
221761_at
219006_at

47069_at
201614_s_at
203162_s_at
220147_s_at
201138_s_at
200770_s_at
219177_at
200873_s_at
201889_at
202246_s_at
209725_at
213701_at
218712_at
216251_s_at
201872_s_at
215645_at
221823_at
212563_at
221535_at
201892_s_at
204610_s_at
219217_at
219143_s_at
200650_s_at
221864_at
218132_s_at
209280_at
217956_s_at
221434_s_at
217373_x_at
202801_at
214794_at
203622_s_at
212482_at
218561_s_at
218710_at
213047_x_at
201519_at
201054_at
206554_x_at
221575_at
209161_at
215380_s_at
214093_s_at
217987_at
59631_at
200953_s_at

214696_at
210802_s_at
212456_at
203286_at
220865_s_at
201024_x_at
203147_s_at
215438_x_at
218398_at
60474_at
208967_s_at
212390_at
52164_at
203065_s_at
214764_at
218082_s_at
214801_at
221931_s_at
208833_s_at
201512_s_at
218012_at
218737_at
201391_at
218762_at
208787_at
203701_s_at
219960_s_at
204133_at
209332_s_at
202138_x_at
33736_at
215412_x_at
206261_at
204808_s_at
212253_x_at
201930_at
209273_s_at
222318_at
201608_s_at
221751_at
218647_s_at
202188_at
203277_at
219031_s_at
208264_s_at
212360_at
202522_at

64900_at
219742_at
201277_s_at
204331_s_at
204186_s_at
201674_s_at
214661_s_at
212416_at
214484_s_at
219095_at
212018_s_at
210528_at
41037_at
202815_s_at
204405_x_at
203431_s_at
209123_at
205004_at
217850_at
201027_s_at
212635_at
200750_s_at
218096_at
215416_s_at
204807_at
203882_at
203362_s_at
201139_s_at
212541_at
221978_at
200903_s_at
219022_at
214662_at
210448_s_at
201410_at
213564_x_at
201998_at
202309_at
217815_at
200037_s_at
216515_x_at
214453_s_at
207843_x_at
218670_at
53968_at
205677_s_at
216996_s_at

206593_s_at
217809_at
212658_at
209100_at
204700_x_at
202232_s_at
204244_s_at
210250_x_at
204027_s_at
214507_s_at
218637_at
210347_s_at
214173_x_at
200662_s_at
213418_at
219037_at
210742_at
201420_s_at
204347_at
219494_at
208985_s_at
204526_s_at
218016_s_at
200840_at
217047_s_at
200658_s_at
217832_at
211576_s_at
212432_at
202126_at
208676_s_at
211933_s_at
204185_x_at
202937_x_at
202934_at
218544_s_at
217755_at
214940_s_at
218897_at
218826_at
200747_s_at
213478_at
202925_s_at
218168_s_at
205455_at
212097_at
222292_at

201490_s_at
203360_s_at
201623_s_at
218738_s_at
215016_x_at
201051_at
221194_s_at
221730_at
219497_s_at
205854_at
208688_x_at
212731_at
218027_at
117_at
218889_at
203624_at
200993_at
209233_at
203244_at
203287_at
214472_at
201947_s_at
207506_at
211059_s_at
202828_s_at
213861_s_at
205115_s_at
204348_s_at
212252_at
200874_s_at
220623_s_at
219131_at
202679_at
220175_s_at
218307_at
201817_at
201075_s_at
201912_s_at
218614_at
205133_s_at
212915_at
209776_s_at
65585_at
210466_s_at
202159_at
218459_at
218932_at

204806_x_at
209825_s_at
204493_at
205539_at
209339_at
212488_at
203380_x_at
207735_at
201160_s_at
217786_at
212038_s_at
91684_g_at
211976_at
203567_s_at
203867_s_at
218331_s_at
219363_s_at
219526_at
221622_s_at
212333_at
212192_at
35436_at
221932_s_at
200995_at
204794_at
221843_s_at
201111_at
209181_s_at
205135_s_at
203721_s_at
200663_at
215165_x_at
211429_s_at
204206_at
201017_at
214355_x_at
202706_s_at
202069_s_at
211971_s_at
212973_at
203610_s_at
202086_at
202435_s_at
206381_at
212546_s_at
205661_s_at
219874_at

200894_s_at
208890_s_at
210561_s_at
209336_at
201797_s_at
218199_s_at
219459_at
212896_at
218305_at
212086_x_at
208933_s_at
218607_s_at
217848_s_at
200659_s_at
203612_at

RAS_MAP (Fig 4)
222227_at
204439_at
205476_at
202859_x_at
205034_at
208583_x_at
218585_s_at
204470_at
38037_at
203821_at
221009_s_at
201890_at
211814_s_at
204102_s_at
204475_at
208891_at
204415_at
208893_s_at
208892_s_at
202086_at
209211_at
204803_s_at
203362_s_at
206176_at
209803_s_at
202422_s_at
205676_at
201930_at
60474_at
213610_s_at

205767_at
205067_at
218796_at
201041_s_at
45633_at
201631_s_at
209189_at
218247_s_at
205290_s_at
222303_at
201294_s_at
205680_at
221489_s_at
221156_x_at
200790_at
218782_s_at
201555_at
205016_at
201694_s_at
204420_at
214753_at
209598_at
211756_at
202436_s_at
206300_s_at
202503_s_at
208476_s_at
214731_at
205266_at
212192_at
209403_at
217997_at
221773_at
201016_at
204011_at
213572_s_at
206581_at
210845_s_at
218871_x_at
212281_s_at
212188_at
200644_at
207717_s_at
218886_at
34408_at
217976_s_at
39402_at

217745_s_at
222036_s_at
215558_at
216342_x_at
220960_x_at
208738_x_at
213156_at
218637_at
202241_at
207655_s_at
205264_at
218647_s_at
204326_x_at
212983_at
202360_at
211764_s_at
203203_s_at
213372_at
218878_s_at
212254_s_at
201693_s_at
202403_s_at
213293_s_at
204127_at
203401_at
201466_s_at
206156_at
202911_at
202330_s_at
221829_s_at
205263_at
203139_at
210561_s_at
205063_at
200749_at
202231_at
208754_s_at
41387_r_at
204405_x_at
212099_at
209457_at
215243_s_at
205068_s_at
215016_x_at
203287_at
202693_s_at
212185_x_at

36711_at
212455_at
217996_at
215434_x_at
203348_s_at
200747_s_at
201328_at
201009_s_at
202935_s_at
218239_s_at
214451_at
203132_at
200608_s_at
219682_s_at
204072_s_at
208711_s_at
217963_s_at
205490_x_at
200919_at
211072_x_at
201202_at
201695_s_at
203177_x_at
213726_x_at
213646_x_at
204028_s_at
212917_x_at
218499_at
200603_at
213271_s_at
202269_x_at
212297_at
202126_at
221775_x_at
219023_at
218738_s_at
211058_x_at
212639_x_at
218096_at
213848_at
219296_at
217738_at
218577_at
203638_s_at
211270_x_at
200047_s_at
218350_s_at

201833_at
217739_s_at
209375_at
212754_s_at
221786_at
209251_x_at
203080_s_at
212286_at
206976_s_at
212267_at
209604_s_at
213881_x_at
200994_at
217850_at
208977_x_at
201152_s_at
201242_s_at
219286_s_at
209433_s_at
204948_s_at
218263_s_at
200594_x_at
218277_s_at
208796_s_at
200773_x_at
201218_at
200873_s_at
208152_s_at
208766_s_at

MYC_MAP (Fig 4)
222227_at
220414_at
202431_s_at
215788_at
208583_x_at
117_at
209776_s_at
201563_at
213418_at
204102_s_at
202086_at
212281_s_at
203023_at
204521_at
203362_s_at
204133_at

212563_at
218590_at
218168_s_at
214059_at
212282_at
204415_at
221586_s_at
216212_s_at
211951_at
203148_s_at
218997_at
205264_at
211576_s_at
203150_at
217140_s_at
212510_at
218481_at
219031_s_at
203119_at
203329_at
205774_at
219497_s_at
218984_at
219006_at
214912_at
214011_s_at
205677_s_at
218305_at
210846_x_at
210347_s_at
209725_at
203622_s_at
219037_at
201014_s_at
210129_s_at
202613_at
214484_s_at
214794_at
201013_s_at
201479_at
219131_at
218976_at
204748_at
209567_at
222305_at
209434_s_at
202823_at

204281_at
218866_s_at
204905_s_at
218882_s_at
221823_at
215726_s_at
202310_s_at
219545_at
218653_at
203882_at
204602_at
221020_s_at
206261_at
50314_i_at
65585_at
214661_s_at
211686_s_at
209433_s_at
204331_s_at
200610_s_at
221634_at
203567_s_at
201516_at
216913_s_at
212488_at
205895_s_at
203196_at
211138_s_at
202690_s_at
213030_s_at
218199_s_at
200875_s_at
201555_at
204175_at
218096_at
210793_s_at
219522_at
221829_s_at
210561_s_at
203325_s_at
200749_at
204807_at
221931_s_at
208264_s_at
201296_s_at
218889_at
217850_at

201491_at
208985_s_at
203568_s_at
209447_at
217164_at
213427_at
204700_x_at
201478_s_at
210230_at
218647_s_at
218637_at
201872_s_at
220688_s_at
205565_s_at
204405_x_at
219178_at
204206_at
205306_x_at
212253_x_at
201139_s_at
217809_at
203287_at
202309_at
200658_s_at
65588_at
202212_at
208754_s_at
220960_x_at
217106_x_at
221843_s_at
201614_s_at
222333_at
208152_s_at
206653_at
218380_at
211921_x_at
209449_at
221899_at
204977_at
217724_at
202800_at
201675_at
210027_s_at
218532_s_at
203405_at
200687_s_at
218670_at

213112_s_at
205437_at
209233_at
202436_s_at
200903_s_at
201674_s_at
204900_x_at
205340_at
202963_at
202138_x_at
218708_at
212846_at
213132_s_at
218512_at
212500_at
212449_s_at
220865_s_at
65133_i_at
205135_s_at
203712_at
206445_s_at
220147_s_at
211725_s_at
212896_at
219960_s_at
218594_at
204593_s_at
210802_s_at
201912_s_at
218544_s_at
210069_at
203162_s_at
218239_s_at
208758_at
212018_s_at
203610_s_at
212541_at
203147_s_at
219503_s_at
215438_x_at
202483_s_at
205133_s_at
208967_s_at
219060_at
212038_s_at
212298_at
219217_at

203244_at
212192_at
202679_at
213188_s_at
208676_s_at
211623_s_at
209864_at
212333_at
201624_at
202126_at
221194_s_at
205330_at
221691_x_at
209143_s_at
205676_at
202435_s_at
203149_at
212898_at
202715_at
218710_at
204808_s_at
205129_at
208896_at
203360_s_at
212254_s_at
214099_s_at
210840_s_at
218398_at
203380_x_at
208696_at
217848_s_at
217862_at
37966_at
212359_s_at
212218_s_at
220251_at
201420_s_at
221536_s_at
205113_at
202761_s_at
218982_s_at
200995_at
205425_at
209440_at
200874_s_at
207826_s_at
204135_at

201054_at
214696_at
202169_s_at
215287_at
214214_s_at
201326_at
202856_s_at
203385_at
209100_at
209161_at
200994_at
221761_at
213701_at
208308_s_at
201490_s_at
218027_at
213581_at
209971_x_at
218460_at
202973_x_at
215380_s_at
208787_at
207622_s_at
218838_s_at
209467_s_at
220233_at
203177_x_at
212434_at
203080_s_at
212701_at
210357_s_at
203401_at
203518_at
202246_s_at
201323_at
203386_at
212915_at
212973_at
218932_at
221970_s_at
203316_s_at
212992_at
215465_at
215016_x_at
209774_x_at
201512_s_at
205076_s_at

217956_s_at
217864_s_at
208682_s_at
209025_s_at
203409_at
200028_s_at
208910_s_at
221535_at
201595_s_at
201327_s_at
201892_s_at
211764_s_at
219250_s_at
219997_s_at
203286_at
220933_s_at
211862_x_at
202364_at
203378_at
203200_s_at
217980_s_at
200898_s_at
206703_at
200772_x_at
200873_s_at
210766_s_at
205658_s_at
201577_at
203798_s_at
218859_s_at
218592_s_at
203431_s_at
202144_s_at
217915_s_at
217963_s_at
35436_at
210976_s_at
200993_at
203810_at
201503_at
203040_s_at
201600_at
217987_at
215501_s_at
212754_s_at
200953_s_at
205068_s_at

203721_s_at
203780_at
221940_at
201138_s_at
204783_at
209247_s_at
218225_at
203737_s_at
215096_s_at
209375_at
202232_s_at
204072_s_at
203462_x_at
217832_at
221622_s_at
204751_x_at
208114_s_at
218556_at
201075_s_at
218364_at
200773_x_at
212922_s_at
212455_at
201277_s_at
200807_s_at
200750_s_at
203651_at
211270_x_at
221276_s_at
218331_s_at
218607_s_at
212175_s_at
218016_s_at
221786_at
201030_x_at
218738_s_at
204809_at
40189_at
220417_s_at
218577_at
218597_s_at
200016_x_at
209626_s_at
211774_s_at
200047_s_at
201129_at
217047_s_at

209902_at
218987_at
201410_at
200992_at
207655_s_at
219110_at
203037_s_at
202918_s_at
218888_s_at
201027_s_at
219363_s_at
210616_s_at
203939_at
218826_at
218088_s_at
209171_at
211954_s_at
218947_s_at
91684_g_at
209317_at
201608_s_at
204180_s_at
202208_s_at
208933_s_at
207076_s_at
212744_at
203584_at
208905_at
211971_s_at
204594_s_at
218263_s_at
201112_s_at
204204_at
202668_at
200662_s_at
201947_s_at
200910_at
201519_at
209682_at
221805_at
202469_s_at
200631_s_at
212097_at
204839_at
209222_s_at
208766_s_at
209296_at

212482_at
212888_at
213295_at
212635_at
33736_at
221813_at
200815_s_at
213802_at
200050_at
203332_s_at
202597_at
208839_s_at
203910_at
201934_at
208926_at
213579_s_at
201817_at
201266_at
204362_at
217900_at
209129_at
212070_at
204542_at
212894_at
200828_s_at
219520_s_at
216483_s_at
201231_s_at
204186_s_at
218082_s_at
220607_x_at
202722_s_at
200738_s_at
204872_at
219459_at
209974_s_at
201832_s_at
202985_s_at
218796_at
201471_s_at
206352_s_at
212169_at
217858_s_at
36936_at
200774_at
202200_s_at
212137_at

200594_x_at
202529_at
200027_at
202776_at
204140_at
212208_at
217812_at
212416_at
222127_s_at
203743_s_at
200063_s_at
204653_at
213503_x_at
210592_s_at
